# MEASURING SOCIOECONOMIC STATUS IN OLDER POPULATIONS: A SCOPING REVIEW AND INTERNATIONAL PERSPECTIVE

**DOI:** 10.1093/geroni/igz038.986

**Published:** 2019-11-08

**Authors:** Gemma F Spiers, Barbara Hanratty, Fiona E Matthews, E Moffatt, A Kingston

**Affiliations:** Newcastle University, Newcastle upon Tyne, United Kingdom

## Abstract

Socioeconomic status (SES) is often measured using indicators that are less relevant to older populations. Building on earlier debates about these issues, an up-to-date, critical review of contemporary evidence and approaches is needed. A key question is how these challenges might vary between countries and different socio-cultural contexts. An international systematic scoping review was undertaken to a) identify which measures of SES have been used in studies of older adults’ health, healthcare utilization and social care utilization, and b) critically appraise the application and validity of these measures in older populations. Systematic searches were conducted in five databases (Medline, Scopus, EMBASE, PsychInfo, Web of Science and Health Management Information Consortium) in May 2018. Studies were eligible if they reported data about the relationship between a measure of SES and self-rated health, healthcare use or social care use for people aged 60+ years, and were published after 2000 in a high-income country (as defined by the Organisation for Economic Cooperation and Development). Sixty-two studies across seventeen countries were included. Measures used included: education (n=41), income (n=37), subjective SES (n=8), occupational or employment (n=10), area deprivation (n=10), combined wealth (n=7), home ownership (n=13), and housing conditions (n=2). A minority (n=7) used a range of proxy variables. The challenges of applying these measures to older populations will be considered. Attention is given to how these challenges may differ by country, whilst considering the added complexities of age, gender and socio-cultural context. Implications for future research on older adults’ health inequalities are discussed.

